# Performance Optimization of Pb_0.97_La_0.03_Sc_0.45_Ta_0.45_Ti_0.1_O_3_ Ceramics by Annealing Process

**DOI:** 10.3390/ma16124479

**Published:** 2023-06-20

**Authors:** Zihan Su, Lingyu Wan, Fenglai Mo, Jiayu Li, Boxun Liu, Chuangjian Liang, Jinsong Xu, Devki N. Talwar, Hang Li, Huilu Yao

**Affiliations:** 1 Center on Nanoenergy Research, Guangxi Colleges and Universities Key Laboratory of Blue Energy and Systems Integration, Carbon Peak and Neutrality Science and Technology Development Institute, School of Physical Science & Technology, Guangxi University, Nanning 530004, China; 2 State Key Laboratory of Featured Metal Materials and Life-Cycle Safety for Composite Structures, Nanning 530004, China; 3 Department of Physics, University of North Florida, Jacksonville, FL 32224, USA

**Keywords:** ferroelectric ceramic, performance optimization, the annealing process

## Abstract

The annealing effects on Pb_0.97_La_0.03_Sc_0.45_Ta_0.45_Ti_0.1_O_3_ (PLSTT) ceramics prepared by the solid-state reaction method are systemically investigated using experimental and theoretical techniques. Comprehensive studies are performed on the PLSTT samples by varying annealing time (AT) from *t* (=0, 10, 20, 30, 40, 50 and 60) h. The properties involving ferroelectric polarization (FP), electrocaloric (EC) effect, energy harvesting performance (EHP) and energy storage performance (ESP) are reported, compared and contrasted. All these features are seen to gradually improve with the increase in AT, and they all reach the climaxed-shaped values and then decrease by further increasing the AT. For *t* = 40 h, the maximum FP (23.2 µC/cm^2^) is attained at an electric field of 50 kV/cm, while the high EHP effects (0.297 J/cm^3^) and positive EC are achieved (for ΔT~0.92 K and ΔS~0.92 J/(K·kg)) at 45 kV/cm. The EHP value of the PLSTT ceramics increased by 21.7% while the polarization value was enhanced by 33.3%. At *t* = 30 h, the ceramics have attained the best ESP value of 0.468 J/cm^3^ with an energy loss of 0.05 J/cm^3^. We strongly believe that the AT plays a crucial role in the optimization of different traits of the PLSTT ceramics.

## 1. Introduction

Ferroelectric materials have recently attracted a great deal of attention due to their growing scientific interests and technological abilities to engineer/design practical devices including energy storage devices, ultrasound transducers, acoustic sensing, modulators, [1,2,3,4,5,6,7,8,9], etc. Among others, Pb-based Pb(Sc_0.5_Ta_0.5_)O_3_-PbTiO_3_ (PST-PT) ceramics as relaxor ferroelectrics have exhibited many unique properties such as high dielectric permittivity over a wide range of temperatures, large electrostrictive strain and ultrahigh piezoelectric coefficients [10,11,12,13,14]. These fundamental traits have clearly distinguished the relaxor ferroelectrics from the so-called diffuse phase transition ferroelectrics where the diffuseness is largely related to the macroscale inhomogeneity and other macroscopic quantities [15]. The basis of typical behavior for relaxor ferroelectrics lies in the interactions of the polarized entities on a nanoscale regime. The materials exhibiting relaxor behavior are typically of a class of oxides commonly known as the “oxygen-octahedra compounds” [16].

For relaxor ferroelectrics, doping has played an important role in effectively improving the performances of different device structures [17,18,19,20]. The effects of lanthanum (La) doping on the microstructure and ferroelectric properties have been extensively studied both in the PST-PT bulk and thin film ceramics. The distribution of La-doping content has exhibited a stronger influence at the microstructure level to modulate the performance of ferroelectric ceramic-based capacitors and piezoelectric devices [21,22,23]. The radius of La^3+^ (1.36 Å) is smaller than that of Pb^2+^ (1.49 Å), and the lattice parameters decrease slightly with the increase in lanthanum content [24]. Appropriate La-doping is known for promoting not only grain growth but also helping to improve the density of the sample and stability of the ferroelectric system. Moreover, it has reduced the coercive field and assisted in achieving outstanding dielectric properties [24,25,26]. As a donor, the substitution of La^3+^ on Pb^2+^ can cause vacancies at the A site of the Pb(Sc_0.5_Ta_0.5_)O_3_ (or ABO_3_) structure. This destroys the transition to a long-range ferroelectric state and forms a new polar cluster so that the ceramics can attain greater energy storage density and efficiency [24,26,27,28,29,30]. Earlier, by doping La into 0.55Pb(Sc_0.5_Ta_0.5_)O_3_-0.45PbTiO_3_ ceramics, Liu et al. [6] studied the major changes in the piezoelectric properties. Maier et al. [31] have also improved the energy storage density by incorporating a small amount of La to affect the structural changes in the ferroelectrics. However, with an additional increase in the La contents, the appearance of pores caused lattice distortions which not only resulted in a substantial increase in the defects but also caused further complications [32,33,34,35]. Moreover, the excess doping of La prevented long-range order of ferroelectricity [36]. The La-doped PST system inhibits the dynamic coupling between eccentric Pb and B-site cations, which is certainly not conducive to further improvement in the performance of their different traits [31]. The development of other technical methods is certainly needed for achieving the optimal performances of ceramic-based device structures.

Many studies [37,38] have demonstrated that the performance of PST-PT material systems for device applications is related not only to the doping but also to the annealing process. For instance, Osbond et al. [37] have examined the (1-x)PST-(x)PT ceramics with different x and compared the changes observed in their dielectric properties after annealing at different temperatures. The authors of Ref. [37] reported that the annealing temperature (AT) plays an important role in the phase transition temperature, dielectric loss, and dielectric constant. Perumal et al. [38] have also studied the energy storage behavior of PZN-PT, PMN-PT, and PZN-PMN-PT (PZN-PbZn_1/3_Nb_2/3_O_3_, PMN-PbMg_1/3_Nb_2/3_O_3_ and PT-PbTiO_3_) materials and revealed their maximum energy storage efficiencies as 45%, 22%, and 71%, respectively. By doping with the La, we have noticed changes in the relaxation properties of PST-PT ceramics to cause a reduction in the coercive field and improve energy storage. 

The purpose of this work is to report the results of systematic annealing processes on the Pb_0.97_La_0.03_(Sc_0.45_Ta_0.45_Ti_0.1_)O_3_ (PLSTT) ceramics prepared by using a conventional solid-state reaction methodology. The microstructure, composition and electrocaloric (EC) properties of the PLSTT ceramics are characterized by exploiting X-ray diffraction (XRD), scanning electron microscopy (SEM), Raman scattering spectroscopy, Energy Dispersive X-ray Spectroscopy (EDS), X-ray Photoelectron Spectroscopy (XPS) and ferroelectric instruments. The effects of annealing time (AT) in hours ranging from *t* (=0, 10, 20, 30, 40, 50, 60 h) are meticulously studied to comprehend the microstructure and macroscopic properties of the PLSTT ceramics. Our systematic and comparative analysis has revealed that AT plays a significant role in the structural characteristics and in effectively improving the polarization performance of the PLSTT ceramics. For an annealing time of 40 h, the study achieved a larger ferroelectric polarization (FP) value of ~23.2 µC/cm^2^ with an electric field of 50 kV/cm, and a higher positive thermoelectric effect (for ΔT~0.92 K, ΔS~0.92 J/(K·kg)) at 45 kV/cm. Moreover, the energy harvesting performance (EHP) also reached a maximum value of ~0.297 J/cm^3^. At the annealing time of 30 h, the ceramic achieves the best energy storage performance (ESP) (0.468 J/cm^3^) with an energy loss of 0.039 J/cm^3^. These outcomes have provided important references for the performance and optimization of PLSTT ceramics.

## 2. Experimental Process

### 2.1. Fabrication

The PLSTT ceramics used in this study were prepared by exploiting a conventional solid-state reaction method considering pure reagents of PbO_2_ (purity ≥ 97%), Ta_2_O_5_ (purity ≥ 99.99%), Sc_2_O_3_ (purity ≥ 99.99%), La_2_O_3_ (purity ≥ 99.9%) and TiO_2_ (purity ≥ 99%) purchased from the Shanghai Aladdin Biochemical Technology Ltd. Co. (Shanghai, China). During the growth process, we added 5% extra of PbO_2_ due to its volatility. The raw materials were ball-milled with zirconia balls in an anhydrous ethanol medium for 12 h. After that, the materials were dried in a ventilated oven set at 120 °C. Subsequent ceramic powders were die-casted and calcined at 900 °C for 2 h and further milled for an additional 12 h. After drying and sieving, the powders were pressed into pellets by using a cold isostatic press at 30 MPa for 7 min. The pellets were first sintered between 1240 °C and 1260 °C for 9 h with a heating rate set at 3 °C/min and subsequently cooled to 700 °C at a cooling rate of 5 °C/min, and finally to room temperature. The sintered samples were then annealed at the rate of 3 °C/min to 1100 °C for *t* (=10, 20, 30, 40, 50, and 60) h.

### 2.2. Characterization

The densities of PLSTT ceramic samples were obtained by Archimedes’ drainage method. The XRD patterns on the ceramics were determined by a PANalytical X′Pert PRO (Almelo, The Netherlands) with Cu Kα radiation (λ = 15.406 nm). The microstructures of PLSTT ceramics were assessed by field emission scanning electron microscopy (SEM, SU8020, Hitachi High-Technologies, Tokyo, Japan). To analyze the ferroelectric properties, the sintered samples were polished down to a thickness of 0.5 mm, then coated with silver paste and calcined for 30 min at 600 °C to fix the Ag electrodes for studying their electrical properties. The hysteresis loops between the polarization value and the electric field strength were tested by using a TF Analyzer 3000, AixACCT, Aachen, Germany.

## 3. Results and Discussion

### 3.1. Structure

**Figure 1:**
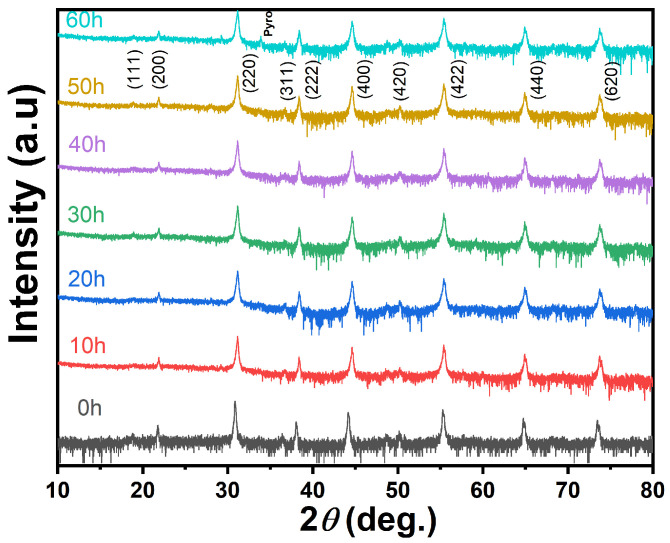
XRD patterns of the PLSTT ceramics with different annealing times under a log (intensity) scale.

For the PLSTT ceramics, we have displayed in Figure 1 the measured XRD diffraction patterns of unannealed and annealed samples with different annealing times *t*. All the diffraction peaks observed in our XRD measurements are indexed by using a cubic perovskite structure with space group Fm3m according to JCPDS 43-0134. While our samples exhibited perovskite structures at T > 1100 °C for different AT, the 60 h AT sample showed the coexistence of perovskite and pyrochlore phase. This observation is consistent with the conclusion drawn in the phase diagram of the lead scandium tantalate-lead titanate (see: Supplementary Figure S1) [39]. Again, our results, when compared with those of Yue et al. [13] find no obvious deviations in the peak positions of the (111), (200), (220), (311), (222), (400), (420), (422), (440) and (620) orientations. It indicated that the annealing process did not cause significant changes in the crystal structure of the PLSTT ceramics. Through the refinement and further analysis of the XRD data, the lattice size, microstrain, dislocation density and lattice parameters of the ceramics are obtained from unannealed and different annealing ATs (see: Supplementary Table S1). This has clearly shown that the unit cell size increases with the increase in AT.

**Figure 2:**
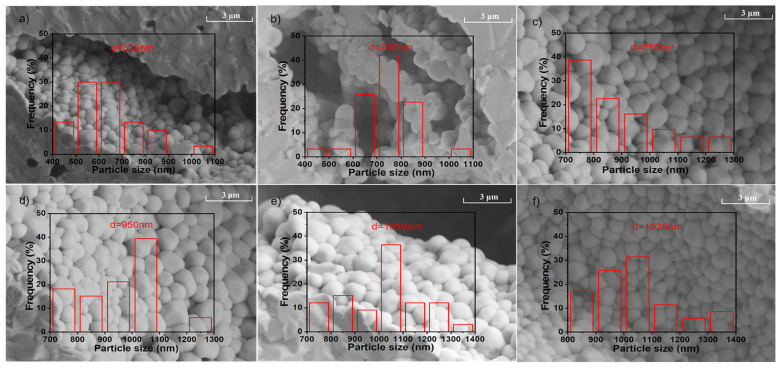
Surface SEM images of PLSTT ceramics after annealing for t hours. (**a**) *t* = 0 h; (**b**) *t* = 10 h; (**c**) *t* = 20 h; (**d**) *t* = 30 h; (**e**) *t* = 40 h and (**f**) *t* = 50 h.

Moreover, our Raman spectroscopic analysis revealed (see: Supplementary Figure S2) that neither new bands appeared nor the old bands disappeared. This study has indicated that consistent with the conclusions drawn by XRD measurements, the phase structure of PLSTT ceramics remained relatively stable. In Figure 2, we have displayed the SEM images of the non-gold-sprayed surface of PLSTT ceramic samples with unannealed and ATs. While the EDS measurements of unannealed PLSTT samples (Supplementary Figure S3) showed a uniform distribution of the elements, the XPS measurements have revealed, however, their exact composition (Supplementary Figure S4). According to the statistical distribution chart (see: Figure 2a–f and Supplementary Figure S5), the average grain sizes in the PLSTT ceramics are 630 nm for unannealed and 740 nm, 880 nm, 950 nm, 1010 nm, 1030 nm, 1170 nm at different ATs (≡10, 20, 30, 40, 50, 60) h, respectively. The results have indicated that the grain size of the ceramics increased with annealing times (see: Supplementary Figure S6). As a result, the microstructure of all ceramics is uniformly compact. This is not only beneficial for improving the insulation performance of ceramics by reducing leakage current but also for lowering the energy loss to enhance the ESP capability.

### 3.2. Ferroelectric Properties

**Figure 3:**
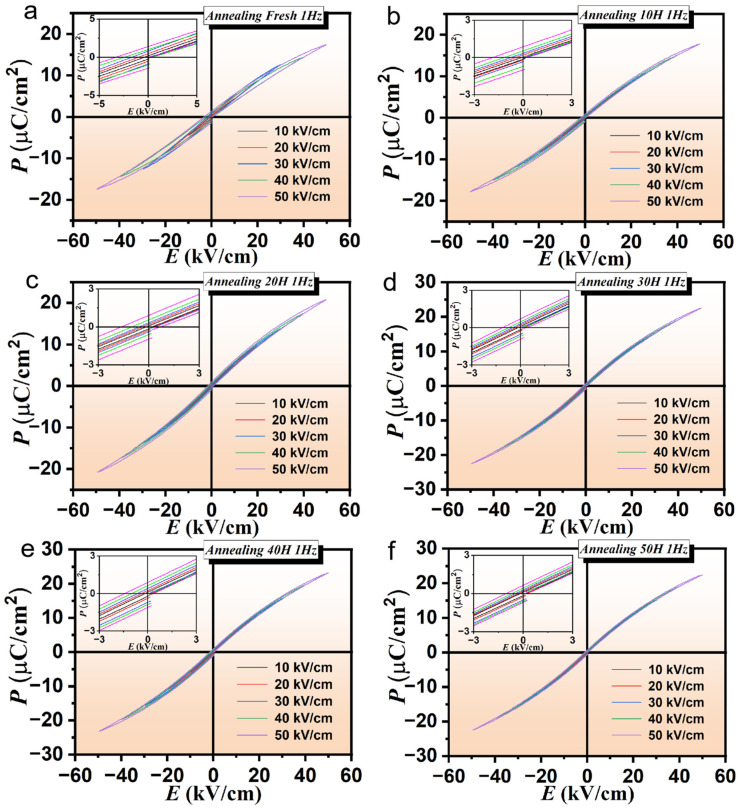
P-E hysteresis loops of PLSTT ceramics with annealing for (**a**) *t* = 0 h; (**b**) *t* = 10 h; (**c**) *t* = 20 h; (**d**) *t* = 30 h; (**e**) *t* = 40 h and (**f**) *t* = 50 h; insets: partial enlarged detail of P-E hysteresis loops (left upper corner) at selected electric fields.

The P-E hysteresis loops of PLSTT ceramic prepared by the conventional solid-state reaction method without annealing at 1 Hz are shown in Figure 3a. The remnant polarization of the hysteresis loop of the sample and the value of the coercive electric field does not change significantly under the electric field from 10 kV/cm to 50 kV/cm, which implied that not only the internal structure of the ceramics is stable and saturated but also consistent with the conclusions drawn by SEM. The sharp edges of the hysteresis loop further indicate that there is no leakage of the electricity. It is worth noting that the FP strength reaches the maximum value of 17.4 µC/cm^2^. The hysteresis loops of PLSTT ceramics under different electric fields (with test condition of room temperature at 1 Hz) and AT between 10 h and 60 h are shown in Figure 3b–f and in the Supplementary Figure S7. 

It can be noticed (cf. Figure 3) that ceramics with different ATs are typically ferroelectrics exhibiting no leakage phenomenon and this is consistent with our analysis by SEM. More interestingly, the maximum FP strength is 17.7, 20.8, 22.5, 23.2, 22.4 and 22.3 µC/cm^2^ for ATs from 10 h to 60 h, respectively. To better compare and further explore the observed phenomena, the maximum polarization values of the unannealed and annealed materials with different ATs (under maximum electric field) are drawn and compared (see: Supplementary Figure S8). It can be noticed that the maximum FP value increases with the increase in AT, and reaches a maximum value when the AT is 40 h. Moreover, the maximum FP value decreases as AT is further increased which fully proved that the relationship between the maximum polarization value of ceramics showed a peak distribution and attained the peak value at about 40 h of annealing time. A further comparison of the maximum FP strength for unannealed and annealed ceramics has revealed that as AT falls between 30 h and 40 h, the polarization strength becomes higher than the unannealed ceramics. This observation fully confirmed the fact that the annealing treatment has the potential to effectively improve the polarization performance of ceramics from the perspective of attaining maximum polarization performance.

### 3.3. Electrocaloric Effect (EC)

**Figure 4:**
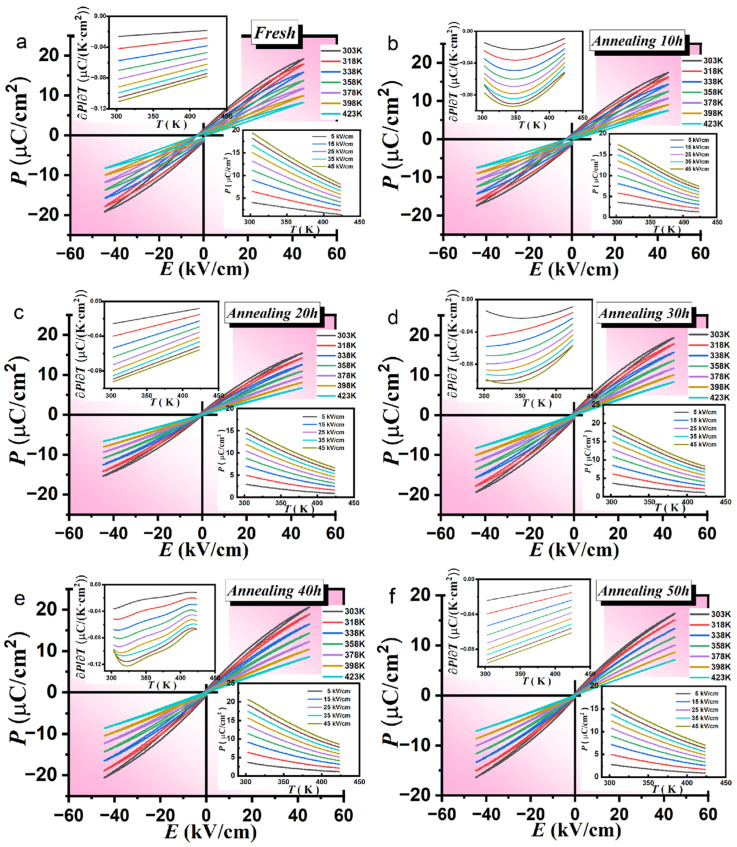
P-E hysteresis loops of PSTT ceramics with annealing for (**a**) *t* = 0 h; (**b**) *t* = 10 h; (**c**) *t* = 20 h; (**d**) *t* = 30 h; (**e**) *t* = 40 h and (**f**) *t* = 50 h; insets: P(T) (right lower corner) and ∂P/∂T (left upper corner) at selected electric fields.

**Figure 5:**
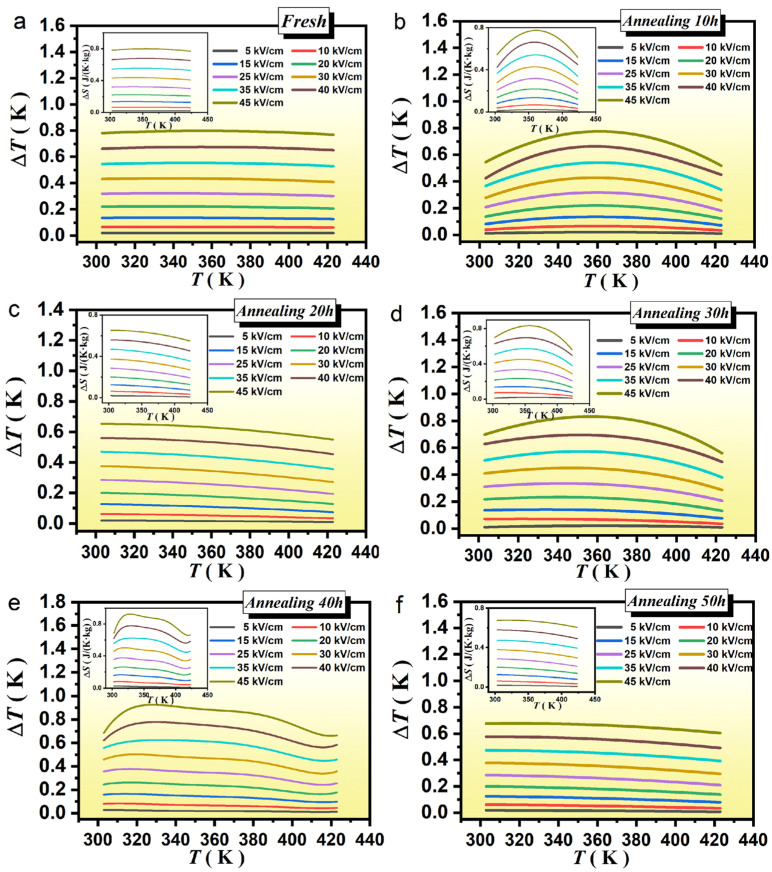
Adiabatic temperature changes ΔT (K) of PLSTT ceramics with annealing for (**a**) *t* = 0 h; (**b**) *t* = 10 h; (**c**) *t* = 20 h; (**d**) *t* = 30 h; (**e**) *t* = 40 h and (**f**) *t* = 50 h; insets: entropy change ΔS with temperature T at different electrical fields.

In Figure 4, we have displayed the P-E hysteresis loops of the PLSTT ceramics from T between 303 K and 423 K at 1 Hz and at 45 kV/cm under different annealing times. The perusal of Figure 4 has clearly revealed that the maximum FP value of ceramic materials for selected electric fields with unannealed and annealed time decreased with increasing temperature. This means that the samples are sensitive to the AT which suggests that they exhibit a positive electrocaloric effect. From Figure 4a, it can be seen that the maximum polarization strength of the unannealed ceramic material reaches the value of 19.1 and 8.2 µC/cm^2^ when the temperature is at T 303 k and 423 K, respectively. Moreover, its variation reaches the value of 10.9 µC/cm^2^, proving that it possesses a certain degree of electrocaloric effect. From Figure 4b–f and Supplementary Figure S9, it is not difficult to find out that the change in the maximum polarization values of ceramic materials annealed for 10 h to 60 h are 9.9, 8.5, 11, 12, 9.3, 8.4 µC/cm^2^, respectively, which also revealed the possibility of attaining an excellent thermoelectric effect. From the above analysis, we have noticed that with the increase in AT, the change in maximum FP increased continuously, reaching a maximum value of 12 µC/cm^2^ at the annealing time of 40 h and with further increasing AT the polarization change gradually decreased. To some extent, this observation has indicated that there is also a peak distribution relationship between the thermoelectric effect and AT. Additionally, by comparing Figure 5b–f and Supplementary Figure S10, we have noticed that the change of polarization value of the ceramics annealed between 30 h and 40 h is better than that of the unannealed ceramics, indicating that the appropriate AT can improve the sensitivity of the samples which is beneficial for expanding the thermoelectric effect.

More importantly, under different electric fields, the relationship curve between the maximum polarization value and temperature is shown in the lower right-hand side of Figure 4. It is easy to find that the maximum FP values increased with the increase in electric field strength and decreased with the increase in temperature. It is worth noting that when AT reaches 40 h and the temperature is 320 K, the relationship curve between the reaction polarization value and the temperature attains a very steep slope. This means that there might be a large thermoelectric effect in its vicinity. For T temperature between 303 K and 343 K the maximum FP value decreased rapidly while decreasing slowly in the temperature range of 343 K to 423 K which indicates that the thermoelectric properties mainly appeared in the temperature range of 303 K to 343 K. The change of ∂P/∂T at selected electric fields is plotted in the upper left insets of Figure 4. Clearly, in the temperature range between 303 K and 343 K, the absolute value of ∂P/∂T is relatively large, and for T between 343 K and 423 K, the absolute value of ∂P/∂T is small, which confirms the above inference. The maximum ∂P/∂T values of PLSTT ceramics under unannealed and different annealing times are −1100, −940, −920, −1040, −1220, −960, and −880 µCm^−2^K^−1^, respectively. Earlier, Crossley et al. [40,41,42] had applied PbSc_0.5_Ta_0.5_O_3_ in multilayer capacitors to achieve the high thermoelectric effect and electrocaloric cooling cycles with true regeneration and measured thermoelectric temperature change by quasi-indirect methods [40,41,42]. Here, we changed the relaxation properties by doping to optimize the material characteristics by annealing and compared the effects of AT on thermoelectric behavior. This study not only proved the correct relationship between ∂P/∂T to achieve peak-shaped distribution but has also fully demonstrated that proper AT can increase the temperature sensitivity of the material, which is conducive to a better electrocaloric effect. All the tests are completed at 1 HZ.

The perusal of Figure 4 has revealed that the maximum FP value decreased with the increase in T, indicating good thermoelectricity. With the increase in AT in PLSTT ceramics, the maximum FP increased gradually, and when the annealing time is 40 h at 45 kV/cm, the maximum polarization value reached a value of 20.6 μC/cm^2^ at T 303 K. The polarization with the greatest variation at 40 h of AT illustrated its relatively excellent performance. This indicated that the appropriate AT improved the EC performance of the samples. The maximum FP value decreased by further increasing the AT. As shown in the lower righthand side of Figure 4, the maximum FP increases with the increase in electric field strength and decreases with the increase in temperature. In a 40 h AT (see: the lower righthand side inset of Figure 4e) the FP changed significantly with T, and there could be a large EC effect in its vicinity. The polarization value decreased sharply with the increase in T from 303 K to 343 K, while between 343 K and 423 K, it decreased slowly (see: upper lefthand side inset of Figure 4e). From the upper lefthand side inset of Figure 4e, the slope between 303 K and 343 K is relatively large but flat between 343 K and 423 K. The other graphs in the upper lefthand side inset of Figure 4, showed relatively flat values of ∂P/∂T.

Under the condition of reversible adiabatic approximation, the Maxwell relationship (∂P/∂T)_E_ = (∂S/∂E)_T_ is assumed to be valid. Thus, the EC effect in PLSTT ceramics is obtained [43,44] by using:

(1) $$∆T=-\frac{1}{\rho }\int_{E1}^{E2}\left(\frac{T}{C}\right)\left(\frac{\partial P}{\partial T}\right)\;EdE$$

(2) $$∆S=-\frac{1}{\rho }\int_{E1}^{E2}\left(\frac{\partial P}{\partial T}\right)\;EdE$$

In Equations (1) and (2), P signifies the maximum polarization at an applied electric field E; T represents the operating temperature and E_1_ and E_2_ stand for the initial and final applied electric field, respectively. In Figure 5 we have displayed the variations of ΔT and ΔS as a function of T for a test frequency of 1 HZ with different electric fields. 

From the simulated features, we have noticed that the values of ΔT and ΔS increased with the increase in E and decreased by increasing T; however, they are all positive > 0. This proved that all ceramic samples exhibited a positive EC effect. Not only the maximum ΔT of each ceramic appeared in the low-temperature region but also the results have illustrated that the materials exhibited a good thermoelectric effect at low T. This proved that ceramics have great prospects for commercial applications. More interestingly, under the selected values of E, the PLSTT ceramics for ATs (≡10, 20, 30, 40, 50 and 60) h have provided maximum ΔT values to be 0.77, 0.65, 0.83, 0.92, 0.68 and 0.62 K, respectively (see: Supplementary Figure S10). The thermoelectric effect gradually increased with the increase in the AT for PLSTT ceramic samples. For AT 40 h, the thermoelectric effect reaches the maximum value (ΔT = 0.92 K, ΔS = 0.92 J/((K)∙kg)). By further increasing AT the thermoelectric effect becomes weaker. This shows that the relationship between the thermoelectric effect and AT still shows a peak distribution, while the maximum value of performance occurs between 30 h and 40 h of annealing. At the same time, ΔT values of unannealed and annealed ceramics at different times compared/contrasted very well. When the annealing time is 30 h and 40 h, the ΔT value is greater than that of unannealed ceramics which means that the proper choice of AT can improve the thermoelectric effects of the PLSTT ceramics.

### 3.4. Energy Harvesting Performance

**Figure 6:**
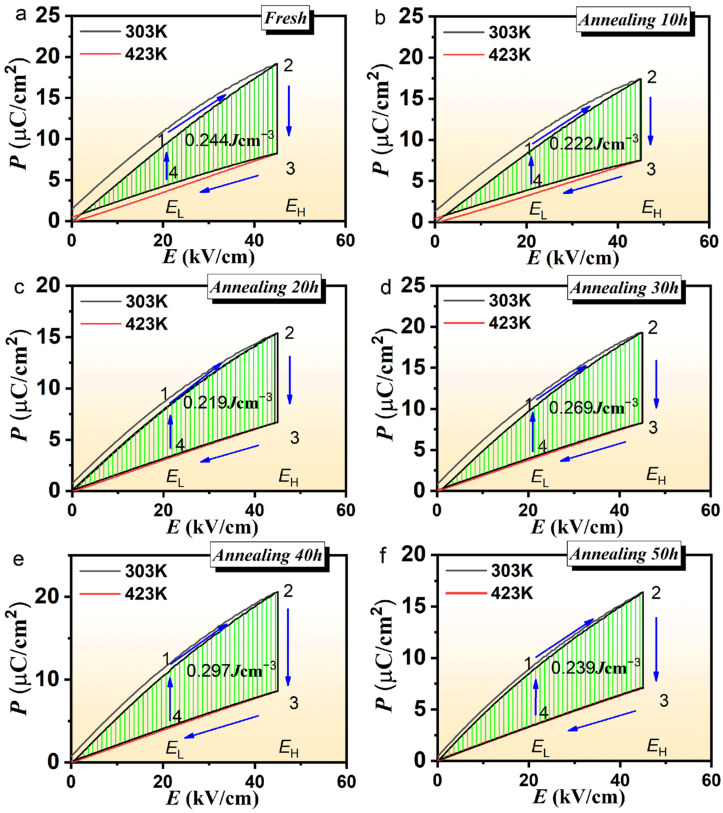
Olsen cycle diagram of pyroelectric energy harvesting of the PLSTT ceramics with annealing for (**a**) *t* = 0 h; (**b**) *t* = 10 h; (**c**) *t* = 20 h; (**d**) *t* = 30 h; (**e**) *t* = 40 h and (**f**) *t* = 50 h.

The Olsen cycle diagram of the PLSTT ceramics is plotted in Figure 6. The process (cf. Figure 6) of 1–2 represents the isothermal increase in the electric field from E_L_ to E_H_ at low temperatures. Process 2–3 means that the PLSTT ceramics are heated from low-temperature P_l_ to high-temperature P_h_ under high electric field E_h_ while process 3–4 signifies the isothermal decrease in the electric field of the ceramic at high-temperature P_h_. The last 4–1 process stands for the cooling of the ceramic under the electric field of E_L_. The energy density captured in the closed loop is equal to the area under the loop of 1→2→3→4 [45,46,47]. While the area can be calculated using Equation (3).

(3) W=∮EdP

Here, the terms E and P represent the electric field intensity and the polarization intensity, respectively. As a result, the energy capture of PLSTT ceramic without annealing is 0.244 J/cm^3^, while it is 0.222 J/cm^3^ when the AT is 10 h, this shows that the appropriate annealing time can help to improve the performance, and the insufficient annealing time will lead to the decrease in the performance. With the increase in annealing time, the captured energy increases and reaches the maximum of 0.297 J/cm^3^ under an annealing time of 40 h, shown in Figure 6a–f and Supplementary Figure S11. However, the captured energy goes down with further increasing of the AT. Overall, it presents a peak distribution with the increase in annealing time. The best energy capture performance occurs during the annealing time between 30 h and 40 h. The energy capture values (0.269 J/cm^3^, 0.297 J/cm^3^) of PLSTT ceramics with an annealing time of 30 h and 40 h are greater than that of the unannealed ceramic, which also implies that proper annealing can enhance the energy capture performance of the ceramic.

### 3.5. Energy Storage Performance

**Figure 7:**
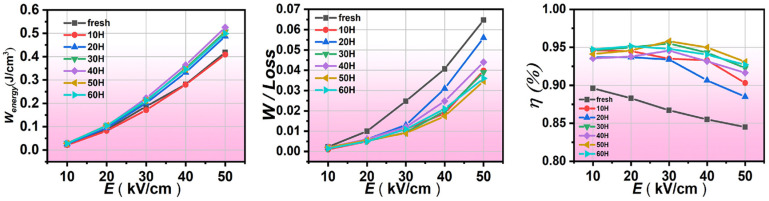
W_energy_, W_loss_, and η of PLSTT ceramics under different annealing time at selected electric fields.

The relationship between the energy storage density W_energy_, energy loss W_loss_, storage efficiency η and electric field intensity of PLSTT ceramics are displayed in Figure 7. The values of W_energy_ and η can be calculated by using Equations (4) and (5), respectively.

(4) $$W=\int_{P_{r}}^{P_{max}}EdP$$

(5) $$\eta =\frac{W_{energy}}{W_{energy}+W_{loss}}\times 100\%$$

In Equations (4) and (5), E is the electric field to cause the change of polarization intensity P, P_max_ is the maximum polarization, and P_r_ is the remnant polarization under the applied electric field. The integration of Equation (4) with a smaller lower limit than the upper limit provides higher energy storage. For material applications, not only large energy storage but also high storage efficiency is required. As can be easily seen from Figure 7, the energy storage density and energy storage loss of PLSTT ceramics increased with the augmentation of the electric field intensity. With the increase in AT, the energy storage density increases and then declines, reaching the maximum value of W_energy_ (= 0.481 J/cm^3^) for 40 h of AT with an electric field of 50 kV/cm. For the AT of 30 h and 40 h, the efficiency change under the selected electric field is not obvious and but always remained above 90%. Under the applied electric field, the energy storage density for 30 h AT becomes lower than that of 40 h AT and the energy storage loss is lower. From the comprehensive view of the three pictures in Figure 7, the energy storage performance is the best when the annealing time is 30 h. 

## 4. Conclusions

In summary, the electrocaloric effect, energy capture performance and energy storage efficiency of PLSTT materials prepared by traditional solid-state reaction methods are significantly improved to varying degrees by annealing treatments. It is found that the comprehensive performance of PLSTT first gradually improves with the increase in annealing time. After reaching a threshold, the performance decreases with the further increase in AT showing a peak shape feature. In particular, after 40 h of annealing, the ceramic material attains a huge thermoelectric performance (ΔT = 0.92 K) and can capture a value of 0.297 J/cm^3^ of ceramic material; when annealed for 30 h, the energy storage performance of the ceramic material is the best, reaching 0.468 J/cm^3^. In addition, the annealing process leads to the formation of larger grains and effectively proves that the grain size of the ceramic material attains an optimal ratio to achieve the best performance of the materials. The annealing process plays an important role in the performance and optimization of ceramic materials. 

## Data Availability

The data that support the findings of this study are available from the corresponding author upon reasonable request.

## Supplementary Materials

The following supporting information can be downloaded at: https://www.mdpi.com/article/10.3390/ma16124479/s1, Figure S1: The phase diagram for the system Pb(Sc_0.5_Ta_0.5_)O_3_-PbTiO_3_(PST-PT); Figure S2: Raman spectra of PLSTT ceramics with different durations [19,48]; Figure S3: Energy dispersive spectroscopy systems (EDS) of the unannealed PLSTT ceramic; Figure S4: X-ray Photoelectron Spectroscopy(XPS) of the unannealed PLSTT ceramic; Figure S5: Surface SEM images of PLSTT ceramics after annealed for 60 h; Figure S6: Grain size variation with annealing time; Figure S7: P-E hysteresis loops of PLSTT ceramics with annealing for 60 h; Figure S8: The contrast of the maximum polarization value of each material under different annealing time; Figure S9: P-E hysteresis loops of PSTT ceramics with annealing for 60 h; insets: P(T) (right lower corner) and ∂P/∂T (left upper corner) at selected electric fields; Figure S10: Adiabatic temperature changes ΔT (K) of PLSTT ceramics with annealing for 60 h; insets: entropy change ΔS(T) with temperature (T) at different electrical fields; Figure S11: Olsen cycle diagram of pyroelectric energy harvesting of the PLSTT ceramics with annealing for 60 h; Table S1: Lattice sizes, dislocation densities, micro strain and lattice parameters of PLSTT ceramics.

## References

[1] Fuflyigin V., Salley E., Vakhutinsky P., Osinsky A., Zhao J., Gergis I., Whiteaker K. (2001). Free-standing films of PbSc_0.5_Ta_0.5_O_3_ for uncooled infrared detectors. Appl. Phys. Lett..

[2] Li J., Liu B., Liang C., Wan L., Wei W., Gao H., Li M., Li Y., Ding W., Qu H. (2022). Fully self-powered electrocaloric cooling/heating with triboelectric nanogenerator. Nano Energy.

[3] Wang Y., Zhang Z., Tomoyasu U., Michael B., Sakyo H., Joseph L., Jamie K., David S. (2020). A high-performance solid-state electrocaloric cooling system. Science.

[4] Alberta E.F., Bhalla A.S. (1996). A Processing and electrical property investigation of the solid solution: (x)Pb(In_1/2_Nb_1/2_)O_3_−(1−x)Pb(Sc_1/2_Ta_1/2_)O_3_. Ferroelectrics.

[5] Brinkman K., Wang Y., Su D., Tagantsev A., Setter N. (2007). The impact of chemical ordering on the dielectric properties of lead scandium tantalate Pb(Sc_1/2_Ta_1/2_)O_3_ thin films. J. Appl. Phys..

[6] Liu H., Wang J., Wang J., Jiang H., Hu X., Dong H. (2000). Dielectric and piezoelectric properties of lanthanum-modified 0.55Pb(Sc_1/2_Ta_1/2_)O_3_−0.45PbTiO_3_ ceramics. J. Eur. Ceram. Soc..

[7] Liu W., Wang G., Cao S., Mao C., Yao C., Cao F., Dong X. (2011). The Phase Transition Behavior of (1−x)Pb(Sc_0.5_Ta_0.5_)O_3_−(x)PbHfO_3_. Ceramics. J. Am. Ceram. Soc..

[8] Frenkel A., Pease D., Giniewicz J., Stern E., Brewe D., Daniel M., Budnick J. (2004). Concentration-dependent short-range order in the relaxor ferroelectric (1−x)Pb(Sc,Ta)O_3_−xPbTiO_3_. Phys. Rev. B.

[9] Liu D., Chen H. (1996). Low-temperature preparation of perovskite Pb(Sc_0.5_Ta_0.5_)O_3_ thin films using MOCVD. Mater. Lett..

[10] Hong Z., Ke X., Wang D., Yang S., Reng X., Wang Y. (2022). Role of point defects in the formation of relaxor ferroelectrics. Acta Mater..

[11] Zhang S., Li F., Jiang X., Kim J., Luo J., Geng X. (2015). Advantages and challenges of relaxor-PbTiO_3_ ferroelectric crystals for electroacoustic transducers—A review. Prog. Mater Sci..

[12] Li F., Cabral M.J., Xu B., Cheng Z., Dickey E.C., LeBeau J.M., Wang J., Luo J., Taylor S., Hackenberger W. (2019). Giant piezoelectricity of Sm-doped Pb(Mg_1/3_Nb_2/3_)O_3_-PbTiO_3_ single crystals. Science.

[13] Yue X., Xiao D., Chao J., Yuan X., Yu G., Xiong X., Wei L., Zhu J. (2004). The crystalline and dielectric properties of (1−x)Pb(Sc_1/2_Ta_1/2_)O_3_–(x)PbTiO_3_ ceramics prepared by one-step-sintering-method. Ceram. Int..

[14] Giniewicz J.R., Bhalla A.S., Cross L.E. (1998). Variable structural ordering in lead scandium tantalate-lead titanate materials. Ferroelectrics.

[15] Choudhary R., Mal J. (2002). Diffuse phase transition in Cs-modified PLZT ferroelectrics. Mater. Sci. Eng. B.

[16] Garten L.M., Burch M., Gupta A.S., Haislmaier R., Gopalan V., Dickey E.C., McKinstry S.T. (2016). Relaxor Ferroelectric Behavior in Barium Strontium Titanate. J. Am. Ceram. Soc..

[17] Correa M., Kumar A., Katiyar R.S. (2007). Investigation of frequency dependent and independent dielectric maxima in relaxor ferroelectric thin films. Appl. Phys. Lett..

[18] Peng B., Fan H., Zhang Q. (2013). A Giant Electrocaloric Effect in Nanoscale Antiferroelectric and Ferroelectric Phases Coexisting in a Relaxor Pb_0.8_Ba_0.2_ZrO_3_ Thin Film at Room Temperature. Adv. Funct..

[19] Peng B., Zhang Q., Gang B., Leighton G., Shaw C., Milne S.J., Zou B., Sun W., Huang H., Wang Z. (2019). Phase-transition induced giant negative electrocaloric effect in a lead-free relaxor ferroelectric thin film. Energy Environ. Sci..

[20] Alpay S.P., Mantese J., Trolier-McKinstry S., Zhang Q., Whatmore R.W. (2014). Next-generation electrocaloric and pyroelectric materials for solid-state electrothermal energy interconversion. Mater. Res. Soc..

[21] Zhang W., Yang J., Wang F., Chen X., Mao H. (2021). Enhanced dielectric properties of La-doped 0.75BaTiO_3_-0.25Bi(Mg_0.5_Ti_0.5_)O_3_ ceramics for X9R-MLCC application. Ceram. Int..

[22] Cao Y., Lin J., Shi Y., Li G., Shi C., Zhu K., Ge G., Chen C., Yan F., Yang W. (2023). High Piezoelectricity in Eco-Friendly NaNbO_3_-Based Ferroelectric Relaxor Ceramics via Phase and Domain Engineering. ACS Appl. Mater. Interfaces.

[23] Liu H., Shi X., Yao Y., Luo H., Li Q., Huang H., Qi H., Zhang Y., Ren Y., Shelly D.K. (2023). Emergence of high piezoelectricity from competing local polar order-disorder in relaxor ferroelectrics. Nat. Commun..

[24] Liu Y., Yang T., Wang H. (2020). Effect of La doping on structure and dielectric properties of PLZST antiferroelectric ceramics. J. Mater. Sci. Mater. Electron..

[25] Xu J., He B. (2019). Structure and properties of La-doped Ba_0.67_Sr_0.33_TiO_3_ environmental ceramics. Mater. Lett..

[26] Li L., Xu M., Zhang Q., Chen P., Wang N., Xiong D., Peng B., Liu L. (2017). Electrocaloric effect in La-doped BNT-6BT relaxor ferroelectric ceramics. Ceram. Int..

[27] Sahoo B., Panda P.K. (2013). Effect of lanthanum, neodymium on piezoelectric, dielectric and ferroelectric properties of PZT. J. Adv. Ceram..

[28] Qiang H., Xu Z. (2020). Enhanced energy storage properties of La-doped Pb_0.99_Nb_0.02_(Zr_0.85_Sn_0.13_Ti_0.02_)_0.98_O_3_ antiferroelectric ceramics. J. Mater. Sci. Mater. Electron..

[29] Ma Z., Zhang Y., Lu C., Qin Y., Lv Z., Lu S. (2018). Synthesis and properties of La-doped PMN–PT transparent ferroelectric ceramics. J. Mater. Sci. Mater. Electron..

[30] Li J., Wang Y., Yang X. (2022). Processing bulk insulating CaTiO_3_ into a high-performance thermoelectric materia. Chem. Eng. J..

[31] Maier B.J., Welsch A.M., Mihailova B., Angel R.J., Bismayer U. (2011). Effect of La doping on the ferroic order in Pb-based perovskite-type relaxor ferroelectrics. Phys. Rev. B.

[32] Levi R.D., Tsur Y. (2005). The Effect of Oxygen Vacancies in the Early Stages of BaTiO_3_ Nanopowder Sintering. Adv. Mater..

[33] Zaman A., Hussain A., Malik R.A., Maqbool A., Kim M.H. (2016). Dielectric and electromechanical properties of LiNbO_3_-modified (BiNa)TiO_3_−(BaCa)TiO_3_ lead-free piezoceramics. J. Phys. D Appl. Phys..

[34] Chan W., Xu Z., Hung T., Chen H. (2004). Effect of La substitution on phase transitions in lead zirconate stannate titanate (55/35/10) ceramics. J. Appl. Phys..

[35] Dan Y., Zou K., Chen G., Yu Y., Zhang Y., Zhang Q., Lu Y., Zhang Q., He Y. (2019). Superior energy-storage properties in (Pb,La)(Zr,Sn,Ti)O_3_ antiferroelectric ceramics with appropriate La content. Ceram. Int..

[36] Xu Z., Dai X., Li J., Viehland D. (1996). Coexistence of incommensurate antiferroelectric and relaxorlike ferroelectric orderings in high Zrcontent Lamodified lead zirconate titanate ceramics. Appl. Phys. Lett..

[37] Osbond P.C., Whatmore R.W. (1993). High dielectric constant ceramics in the PbSc_0.5_Ta_0.5_O_3_-PbZrO_3_ and PbSc_0.5_Ta_0.5_O_3_-PbTiO_3_ systems. J. Mater. Sci..

[38] Perumal R.N., Athikesavan V. (2019). Investigations on electrical and energy storage behaviour of PZN-PT, PMN-PT, PZN–PMN-PT piezoelectric solid solutions. J. Mater. Sci. Mater. Electron..

[39] Burton B.P., Cohen R.E. (1995). Nonempirical calculation of the Pb(Sc_0.5_Ta_0.5_)O_3_-PbTiO_3_ quasibinary phase diagram. Phys. Rev. B.

[40] Crossley S., Nair B., Whatmore R.W., Moya X., Mathur N.D. (2019). Eletrocaloric Cooling Cycles in Lead Scandium Tantalate with True Regeneration via Field Variation. Phys. Rev. X.

[41] Nair B., Usui T., Crossley S., Kurdi S., Guzman-Verri G.G., Moya X., Hirose S., Mathur N.D. (2019). Large electrocaloric effects in oxide multilayer capacitors over a wide temperature range. Nature.

[42] Crossley S., Whatmore R.W., Mathur N.D., Moya X. (2021). Quasi-indirect measurement of electrocaloric temperature change in PbSc_0.5_Ta_0.5_O_3_ via comparison of adiabatic and isothermal electrical polarization data. APL Mater..

[43] Peng B., Fan H., Zhang Q. (2013). High Tunability in (111)-Oriented Relaxor Pb_0.8_Ba_0.2_ZrO_3_ Thin Film with Antiferroelectric and Ferroelectric Two-Phase Coexistence. J. Am. Ceram. Soc..

[44] Peng B., Zhang Q., Lyu Y., Liu L., Lou X., Shaw C., Huang H., Wang Z. (2018). Thermal strain induced large electrocaloric effect of relaxor thin film on LaNiO_3_/Pt composite electrode with the coexistence of nanoscale antiferroelectric and ferroelectric phases in a broad temperature range. Nano Energy.

[45] Olsen R.B., Briscoe J.M., Bruno D.A., Butler W.F. (1981). A pyroelectric energy converter which employs regeneration. Ferroelectrics.

[46] Olsen R.B., Brown D.D. (1982). High efficieincy direct conversion of heat to electrical energy-related pyroelectric measurements. Ferroelectrics.

[47] Olsen R.B., Bruno D.A., Briscoe J.M., Dullea J. (1984). Cascaded pyroelectric energy converter. Ferroelectrics.

[48] Kreisel J., Bouvier P., Maglione M., Dkhil B., Simon A. (2004). High-Pressure Raman Investigation of the Pb-Free Relaxor BaTi_0.65_Zr_0.35_O_3_. Phys. Rev. B.

