# Supplementary material for: Performance Optimization of Pb0.97La0.03Sc0.45Ta0.45Ti0.1O3 Ceramics by Annealing Process
Source: Materials (Basel). 2023 Jun 20;16(12):4479. doi: 10.3390/ma16124479 (PMC10305350; doi:10.3390/ma16124479)
Supplement: Supplementary file 1 [file materials-16-04479-s001.zip › materials-2415603-supplementary.pdf]

Performance optimization of $Pb_{0.97}La_{0.03}Sc_{0.45}Ta_{0.45}Ti_{0.1}O_3$ ceramics by annealing process

Zihan Su [1,2], Lingyu Wan [1,2,*], Fenglai Mo[1,2], Jiayu Li[1,2], Boxun Liu[1,2], Chuangjian Liang[1,2], Jinsong Xu[1,2], Devki N. Talwar[3], Hang Li[1,2], Huilu Yao[1,2]

* **Correspondence: lyw2017@gxu.edu.cn; Tel.: +8613878156436;**

[1] Center on Nanoenergy Research, Guangxi Colleges and Universities Key Laboratory of Blue Energy and Systems Integration, carbon peak and neutrality science and technology development institute, School of Physical Science & Technology, Guangxi University, Nanning 530004, China

[2] State Key Laboratory of Featured Metal Materials and Life-cycle Safety for Composite Structures, Nanning 530004, China

[3] State Department of Physics, University of North Florida, Jacksonville, FL 32224, USA

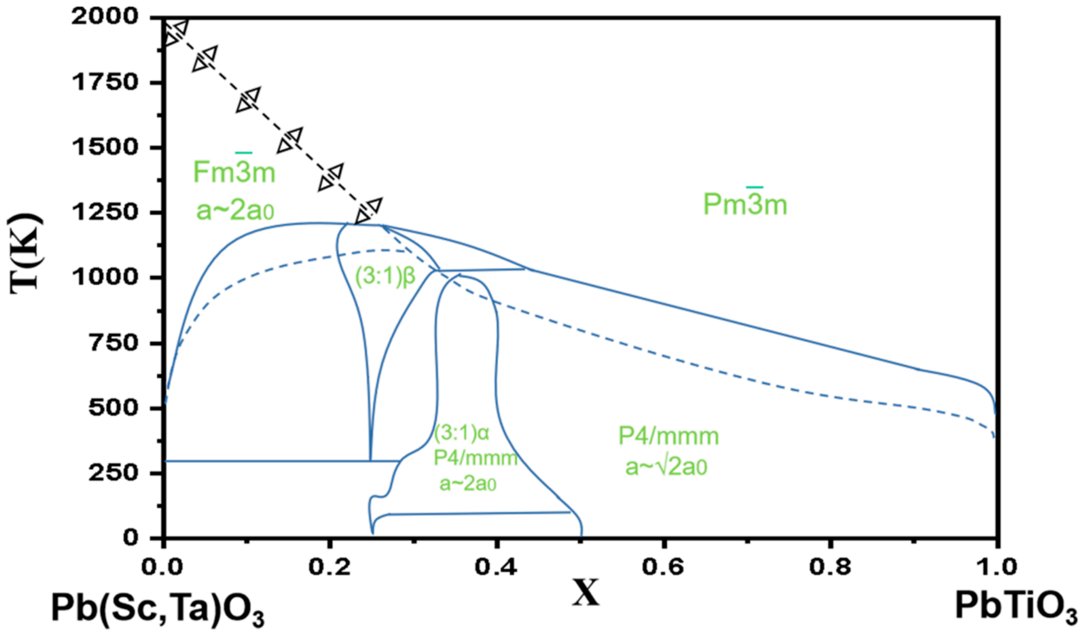

Figure S1. The phase diagram for the system $Pb(Sc_{0.5}Ta_{0.5})O_3$-$PbTiO_3$(PST-PT).

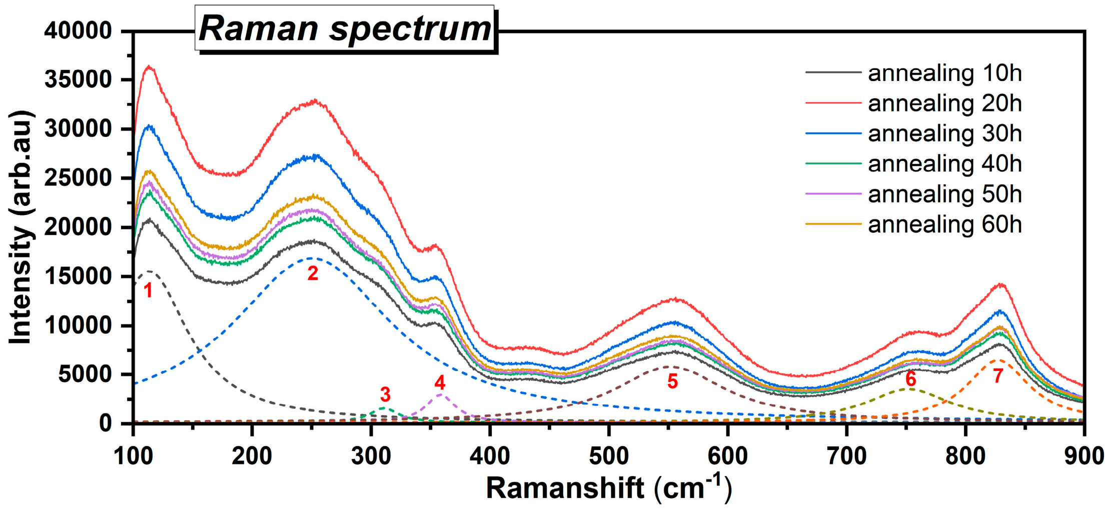

Figure S2. Raman spectra of PLSTT ceramics with different durations.

To reveal the possible effect of the annealing process on the lattice structure of PLSTT ceramics, Raman spectroscopy tests were performed and the results were well fitted to 7 characteristic peaks. Among them, the peak near 100 cm^{-1} is mainly caused by the vibration of the a-position atom in the perovskite structure[1], and the peak near 200–250cm^{-1} is not only related to the vibration of the asymmetric O phonon in the orthogonal phase, but also to the vibration in the rhombohedral phase.

The peak near 300–360cm^{-1} is mainly caused by the asymmetry (TiO_2, Sc_2O_3, Ta_2O_5 phonon vibration) in the tetragonal/orthogonal phase [19,48]; the peak near 550 cm^{-1} is mainly

caused by the vibration of TiO_2/Sc_2O_3 symmetrical stretching in the tetragonal twisted polar octahedral cluster; and the peak near 750 cm^{-1} is mainly the vibration of polar clusters, and its appearance is considered to be a relaxation characteristic.

Table S1. Lattice sizes, dislocation densities, micro strain and lattice parameters of PLSTT ceramics

The annealing time (Hour)	The lattice size (nm)	The dislocation density ($\delta\times10^{-3}nm^{-2}$)	The micro strain ($\varepsilon\times10^{-3}$)	lattice parameters (a=b=c)
0	56	0.36	1.75	8.09
10	58	0.32	1.67	8.10
20	61	0.30	1.59	8.11
30	62	0.28	1.53	8.11
40	63	0.27	1.50	8.12
50	70	0.23	1.39	8.12
60	72	0.22	1.34	8.13

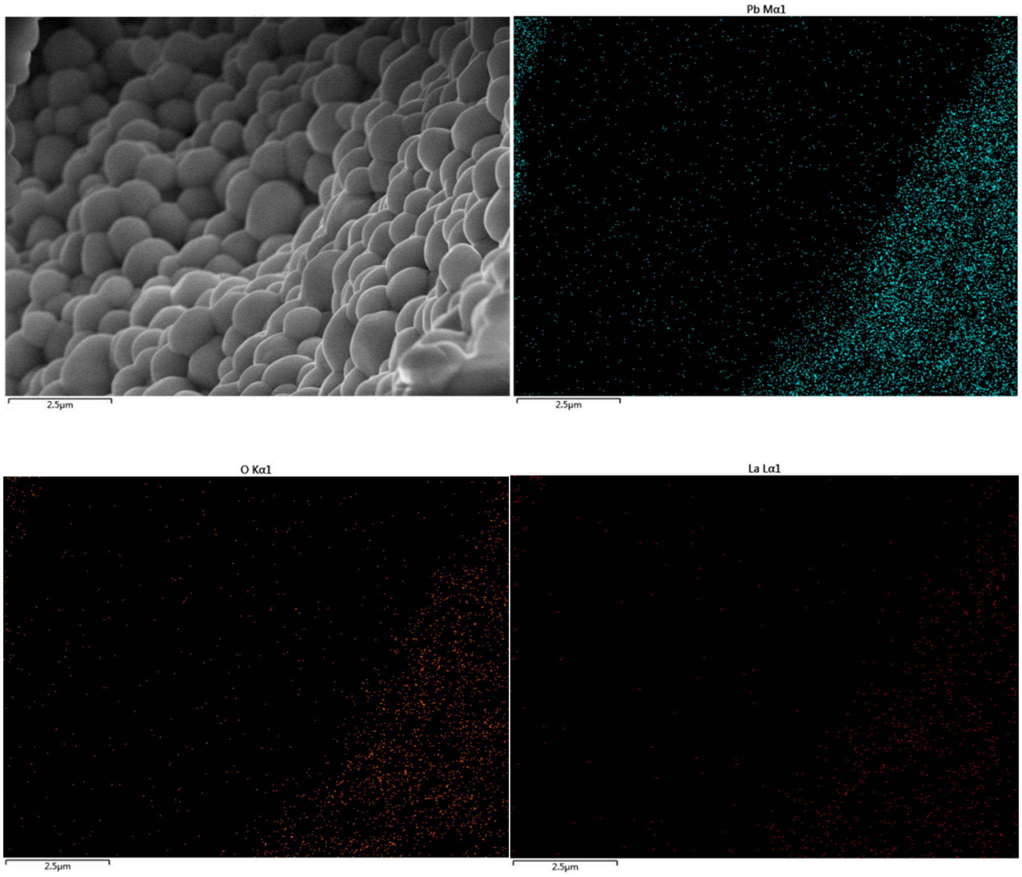

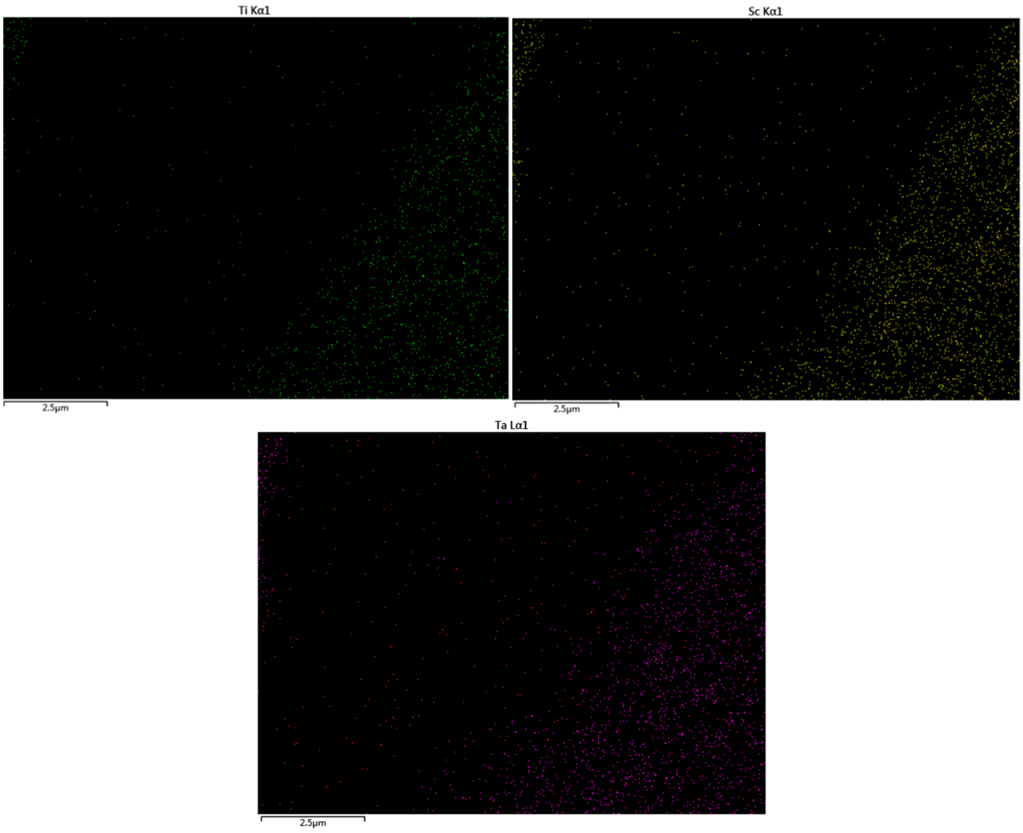

Figure S3. Energy dispersive spectroscopy systems (EDS) of the unannealed PLSTT ceramic.

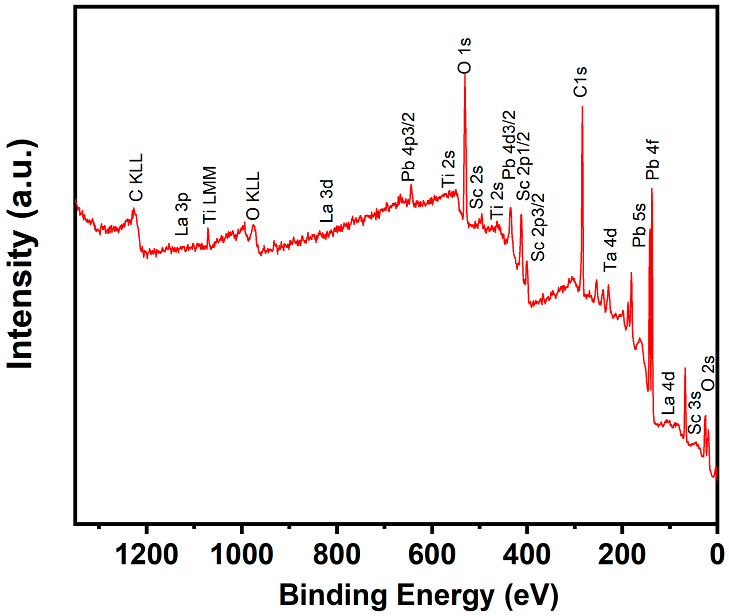

Figure S4. X-ray Photoelectron Spectroscopy(XPS) of the unannealed PLSTT ceramic.

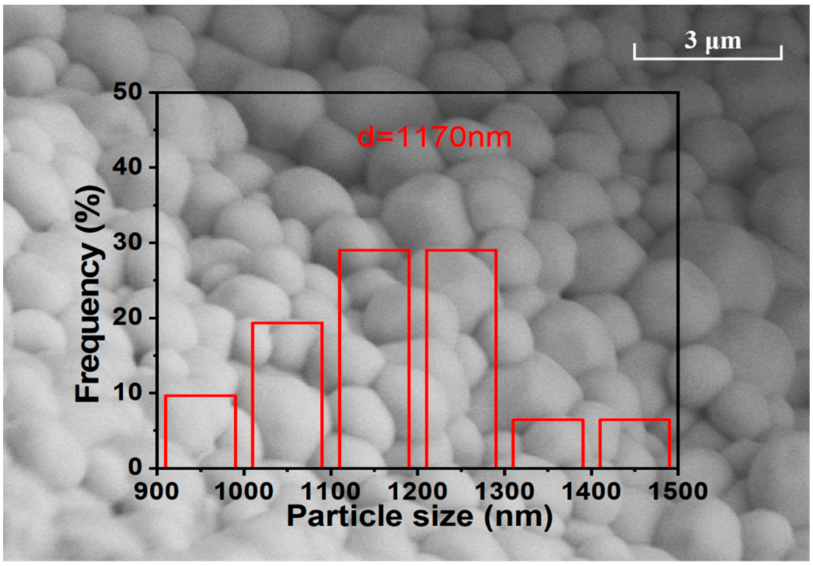

Figure S5. Surface SEM images of PLSTT ceramics after annealed for 60 h.

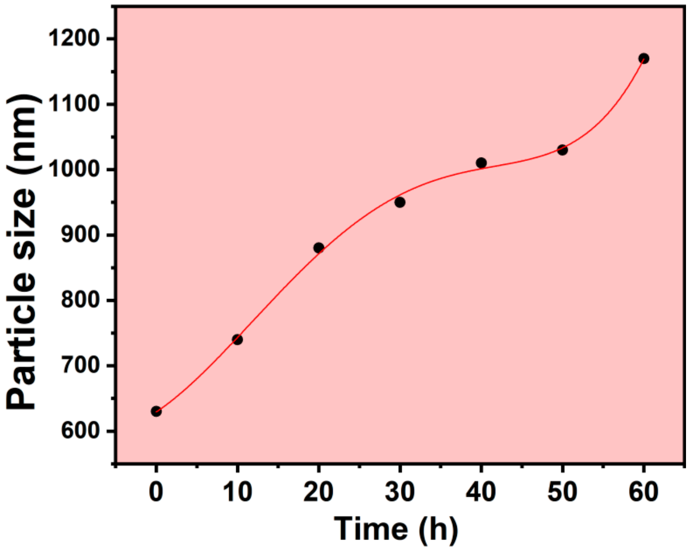

Figure S6. Grain size variation with annealing time.

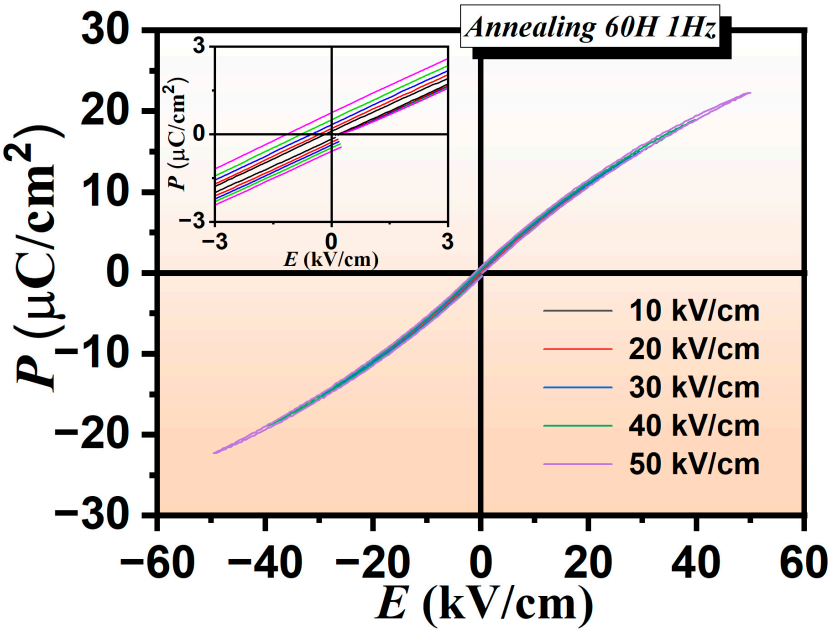

Figure S7. P-E hysteresis loops of PLSTT ceramics with annealing for 60 h; insets: partial enlarged detail of P-E hysteresis loops (left upper corner) at selected electric fields.

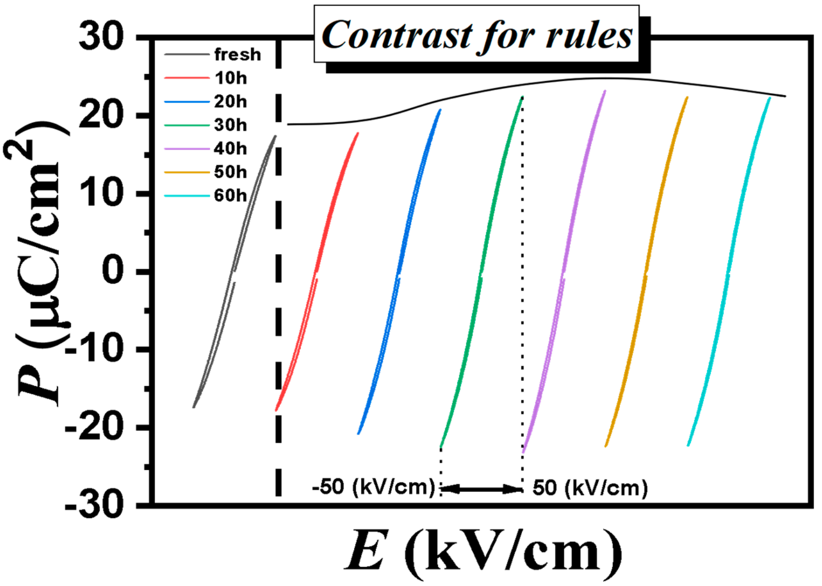

Figure S8. The contrast of the maximum polarization value of each material under different annealing time.

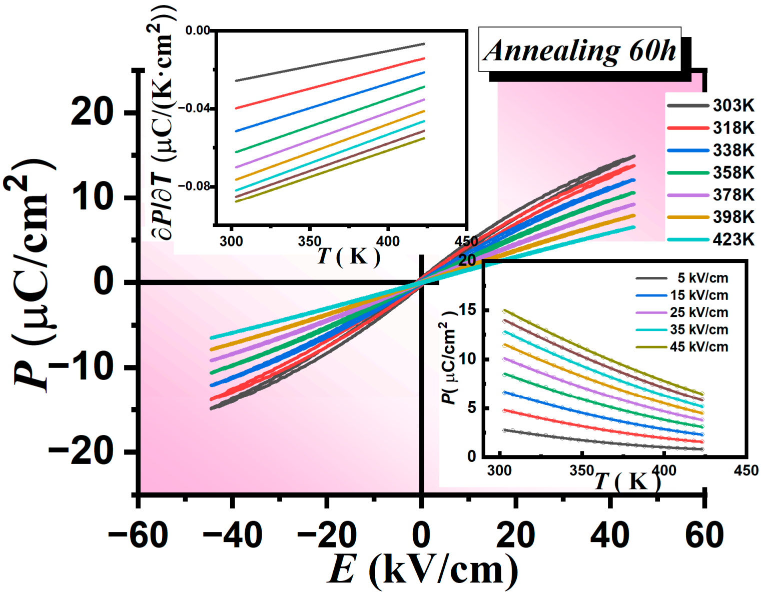

Figure S9. P-E hysteresis loops of PSTT ceramics with annealing for 60 h; insets: P(T) (right lower corner) and ∂P/∂T (left upper corner) at selected electric fields.

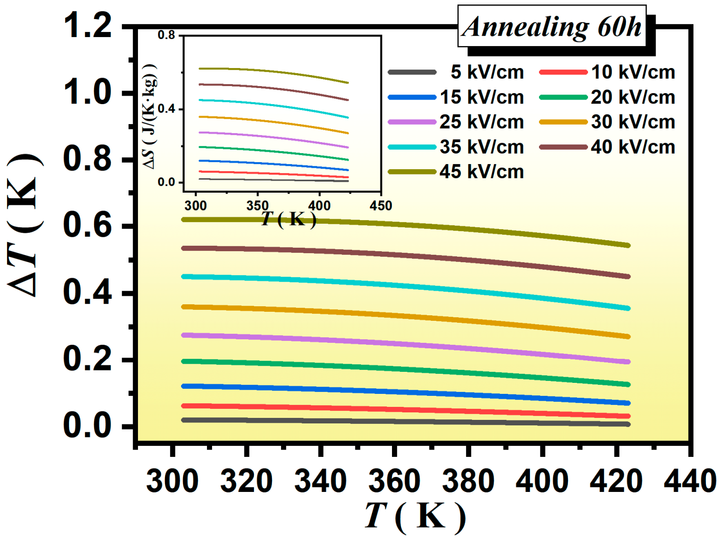

Figure S10. Adiabatic temperature changes ΔT (K) of PLSTT ceramics with annealing for 60 h; insets: entropy change ΔS(T) with temperature (T) at different electrical fields.

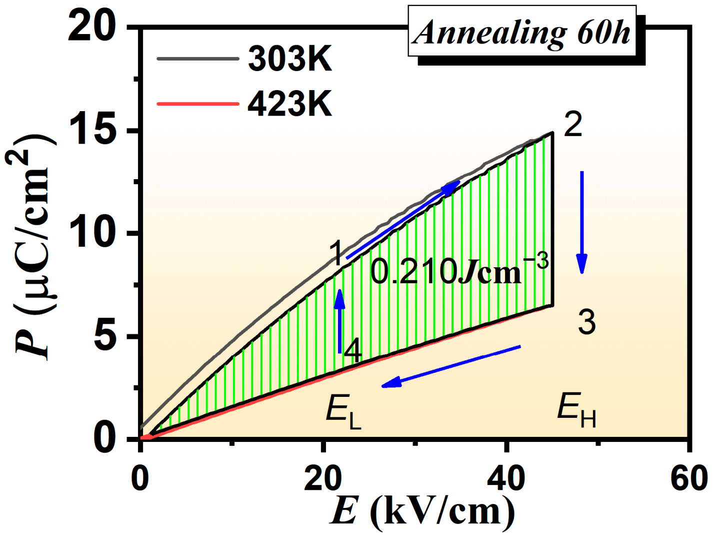

Figure S11. Olsen cycle diagram of pyroelectric energy harvesting of the PLSTT ceramics with annealing for 60 h.

[19] Peng, B.; Zhang, Q.; Gang, B.; Leighton, G.; Shaw, C.; Milne, S.J.; Zou, B.; Sun, W.; Huang, H.; Wang, Z. Phase-transition induced giant negative electrocaloric effect in a lead-free relaxor ferroelectric thin film. *Energ. Environ. Sci.* **2019,** 12, 1708-1717, doi:10.1039/c9ee00269c.

[48] Kreisel, J.; Bouvier, P.; Maglione, M.; Dkhil, B.; Simon, A. High-Pressure Raman Investigation of the Pb-Free Relaxor $BaTi_{0.65}Zr_{0.35}O_3$. *Phys. Rev. B* **2004**, 69, 092104, doi:10.1103/PhysRevB.69.092104.
